# The VITAH Trial Vitamin D supplementation and cardiac autonomic tone in hemodialysis: a blinded, randomized controlled trial

**DOI:** 10.1186/1471-2369-15-129

**Published:** 2014-08-06

**Authors:** Michelle C Mann, Derek V Exner, Brenda R Hemmelgarn, David A Hanley, Tanvir C Turin, Jennifer M MacRae, Sofia B Ahmed

**Affiliations:** 1 Department of Medicine, University of Calgary, 1403-29th St. NW, Room C210D, T2N 2 T9 Calgary, Alberta, Canada; 2 Libin Cardiovascular Institute of Alberta, Calgary, Alberta, Canada; 3 Department of Community Health Sciences, University of Calgary, Calgary, Alberta, Canada; 4 Osteoporosis and Metabolic Bone Disease Centre, Calgary, Alberta, Canada

**Keywords:** Chronic kidney disease, Vitamin D, Ergocalciferol, Alfacalcidiol, Autonomic nervous system, Cardiovascular risk, Clinical trial

## Abstract

**Background:**

Patients with end-stage kidney disease (ESKD) have a high rate of mortality and specifically an increased risk of sudden cardiac death (SCD). Impaired cardiac autonomic tone is associated with elevated risk of SCD. Moreover, patients with ESKD are often vitamin D deficient, which we have shown may be linked to autonomic dysfunction in humans. To date, it is not known whether vitamin D supplementation normalizes cardiac autonomic function in the high-risk ESKD population. The VITamin D supplementation and cardiac Autonomic tone in Hemodialysis (VITAH) randomized trial will determine whether intensive vitamin D supplementation therapies improve cardiac autonomic tone to a greater extent than conventional vitamin D supplementation regimens in ESKD patients requiring chronic hemodialysis.

**Methods**/**Design:**

A total of 60 subjects with ESKD requiring thrice weekly chronic hemodialysis will be enrolled in this 2x2 crossover, blinded, randomized controlled trial. Following a 4-week washout period from any prior vitamin D therapy, subjects are randomized 1:1 to intensive versus standard vitamin D therapy for 6 weeks, followed by a 12-week washout period, and finally the remaining treatment arm for 6 weeks. Intensive vitamin D treatment includes alfacalcidiol (activated vitamin D) 0.25mcg orally with each dialysis session combined with ergocalciferol (nutritional vitamin D) 50 000 IU orally once per week and placebo the remaining two dialysis days for 6 weeks. The standard vitamin D treatment includes alfacalcidiol 0.25mcg orally combined with placebo each dialysis session per week for 6 weeks. Cardiac autonomic tone is measured via 24 h Holter monitor assessments on the first dialysis day of the week every 6 weeks throughout the study period. The primary outcome is change in the low frequency: high frequency heart rate variability (HRV) ratio during the first 12 h of the Holter recording at 6 weeks versus baseline. Secondary outcomes include additional measures of HRV. The safety of intensive versus conventional vitamin D supplementation is also assessed.

**Discussion:**

VITAH will determine whether an intensive vitamin D supplementation regimen will improve cardiac autonomic tone compared to conventional vitamin D supplementation and will assess the safety of these two supplementation regimens in ESKD patients receiving chronic hemodialysis.

**Trial registration:**

ClinicalTrials.gov, NCT01774812

## Background

More than 40,000 Canadians live with end-stage kidney disease (ESKD)—roughly 1 out of every 1,000 people, a number that has tripled over 20 years – and require life-sustaining dialysis or transplantation [1]. Patients with ESKD have a 20% annual mortality rate and an age-specific cardiovascular death rate that is 10-100x higher than the general population [2]. Sudden cardiac death (SCD) accounts for more than a quarter of deaths in the hemodialysis population, making it the leading cause of death [2]. However, as is repeatedly reflected by negative trials evaluating the effects of treatment of traditional cardiovascular disease risk factors in patients with ESKD [3-6], these deaths are not associated with typical coronary artery disease, suggesting alternative mechanisms for SCD in ESKD. As such, the pathophysiology of SCD in the ESKD population is complex and believed to require the interaction between a transient event (i.e. intermittent hemodialysis) and underlying substrate. This process induces electrical instability and ventricular arrhythmias followed by hemodynamic collapse. Two factors specific to ESKD patients that have emerged as potential contributors to this SCD-susceptible state are altered mineral metabolism, including vitamin D deficiency, and impaired cardiac autonomic tone [7-10].

### Cardiac autonomic tone

Cardiac autonomic tone is derived from beat to beat measurements of the time intervals between successive QRS intervals, specifically RR intervals. Measurement of HRV provides direct insight into abnormalities of the autonomic nervous system, which consists of the sympathetic nervous system (SNS) and the parasympathetic (vagal) nervous system (PNS) limbs [7,11,12]. HRV assesses the dynamic interaction and balance between the SNS and PNS at the level of the heart, overall providing a measure of cardiac autonomic competence [11,12]. The *a priori* main outcomes of interest are measures of the frequency domain as these parameters are direct measures of cardiac autonomic tone [11] and are most commonly described in the ESKD study population [10,13-15]. No one parameter of HRV has been established as superior in regards to their predictive ability as surrogate markers for adverse cardiovascular events [7,16-19].

Decreased HRV is associated with an increased risk of arrhythmias and cardiac death in both the general population [7] and patients with ESKD on dialysis [8,9,12-15]. Further, alterations in HRV occur more frequently in dialysis patients and corresponds with an increased risk of SCD and cardiovascular mortality [9,13]. In a study of 30 ESKD patients, 53% had autonomic dysfunction, in which 40% was isolated to the vagal (PNS) limb and 13% had combined sympathetic and vagal dysfunction [20]. Similarly, HRV findings in a study of 239 hemodialysis patients demonstrated drastic sympathetic over-activity and vagal withdrawal [15]. Another study of 383 patients with ESKD on hemodialysis found that impaired cardiac autonomic tone was independently associated with an increased risk of all-cause and cardiovascular death after adjustment for traditional cardiovascular risk factors [8]. Although HRV is a surrogate measurement, it has been established as an important and validated, non-invasive measure of cardiovascular-related mortality risk in the ESKD population [12,13,20,21]. Consequently, interventions enhancing measures of HRV, and therefore influencing cardiac autonomic control, could potentially have considerable survival benefits in the high-risk ESKD patient population.

### Vitamin D

There are multiple pharmacological analogues and formulations of vitamin D available for clinical use [22,23]. To be effective in disorders resulting from vitamin D deficiency, nutritional sources of vitamin D (cholecalciferol, vitamin D_3_; and ergocalciferol, vitamin D_2_) must undergo two enzymatic hydroxylations: 1) in the liver to 25-hydroxyvitamin D (25OHD); 2) in the kidney, 1α-hydroxylase converts 25OHD to the biologically active form of vitamin D, 1,25-dihydroxycholecalciferol (calcitriol) or 1,25- dihydroxyergocalciferol [22,23]. The liver hydroxylation is largely substrate driven, but the kidney’s 1α-hydroxylase is very tightly regulated by hormones (e.g. parathyroid hormone) and concentrations of calcium and phosphate ions [22,23]. In the VITAH trial, we use the following terminology for the specific vitamin D analogues; nutritional vitamin D therapy is provided in the form of ergocalciferol, and alfacalcidiol, is provided as the active (activated) vitamin D derivative.

In ESKD, progressive loss of functional kidney activity reduces the availability of 1α-hydroxylase within the renal tissue, which in turn reduces 1,25-dihydroxyvitamin D_3_ production [24]. ESKD patients are also commonly deficient in 25OHD due to decreased nutritional intake from intensive dietary restrictions, lack of sun exposure due to decreased mobility, and dysregulated mineral metabolism [24].

In general, vitamin D deficiency is common and associated with worse cardiovascular outcomes in both the healthy [25] and ESKD [26] populations. Furthermore, low vitamin D levels are associated with increased risk of SCD in both non-ESKD [27] and ESKD patients [28,29]. In relation to cardiovascular pathophysiology, vitamin D has been shown to influence cardiac contractility and myocardial calcium homeostasis in humans [30-33]. Furthermore, a small study of hemodialysis patients showed that treatment with activated vitamin D reduced the QT interval on electrocardiography [34]. Recently, we have not only shown that low vitamin D levels are associated with vascular physiology that increases cardiovascular risk [35], but also that poor vitamin D status is associated with an impaired ability to maintain cardiac autonomic tone in response to endogenous angiotensin II in healthy humans [36], a hormone that is chronically upregulated in the ESKD population. Furthermore, we have also demonstrated that vitamin D supplementation is associated with *normalization* of autonomic tone in response to an acute angiotensin II stressor in healthy subjects [37]. As vagal activity is markedly reduced in hemodialysis patients [15], it is possible that vitamin D supplementation could improve the cardiac autonomic response to stressors such as hemodialysis. Regardless, the impact of vitamin D therapy in the ESKD population, and on other surrogate clinical measures of cardiovascular risk has not been assessed. In an observational study, serum 25OHD predicted total and cardiovascular mortality in incident ESKD patients, but this association was abolished in patients provided therapy with calcitriol or its active analogues [26]. This novel observation suggests that intensive vitamin D therapy inclusive of 1,25-dihydroxyvitamin D_3_ analogues as well as other vitamin D derivatives may provide a more significant therapeutic and survival benefit within the severely deficient ESKD population, and thus provides the basis for assessing the therapeutic role of intensive and conventional vitamin D supplementation regimens in altering cardiac autonomic tone, as in the VITAH trial.

Evidence to date supports the safety profile of vitamin D therapy. Vitamin D deficiency is the norm in hemodialysis patients and as such, vitamin D supplementation should result in significant increase vitamin D levels. Secondly, given there is significant controversy regarding the target 25-hydroxyvitamin D level in both the general and chronic kidney disease population [38,39], we aimed to determine the effect of delivered dose of vitamin D, rather than level of vitamin D, on cardiac autonomic tone. Lastly, though there is significant practice variation, the activated and nutritional vitamin D doses chosen for the study reflect those recommended in and typically prescribed to the end-stage kidney disease population on hemodialysis [38].

Despite the high prevalence of vitamin D deficiency in ESKD, current guidelines regarding correction of vitamin D status are widely acknowledged to be opinion-based and derived from biochemical endpoints [38]. The nutritional vitamin D product (ergocalciferol) and dose proposed within this protocol is identical to that suggested by guidelines for treatment in the ESKD population [38,40]. Furthermore, treatment with other nutritional forms of vitamin D_3_ at a dose of 15,000 IU/day × 1 month [41] and 40,000 IU/day over 28 weeks, followed by 10,000 IU/day × 12 weeks [42] has been shown to be safe. A main clinical concern with activated vitamin D supplementation in the ESKD population is hypercalcemia, hyperphosphatemia and reduction in parathyroid hormone levels. This protocol employs the lowest possible dose of activated vitamin D (alfacalcidiol) available to minimize significant fluctuations in these biochemical values, though the relative importance of these changes remains unquantified within the literature [43].

In summary, the evidence to guide treatment that may minimize SCD and cardiovascular risk in ESKD is extremely limited. The VITamin D supplementation and cardiac Autonomic tone in Hemodialysis (VITAH) study is the first clinical trial to assess whether various vitamin D therapies, specifically ingestion of the combination of activated and nutritional vitamin D compared to activated vitamin D alone, influence cardiac autonomic tone and therefore cardiovascular risk in ESKD patients requiring hemodialysis.

## Methods

### Study aims

The primary aim of the VITAH trial is to determine whether 6 weeks of intensive vitamin D supplementation, consisting of alfacalcidiol 0.25mcg orally at every dialysis session combined with ergocalciferol 50,000 IU orally once per week and placebo the remaining 2 weekly dialysis sessions, will influence HRV assessment of cardiac autonomic tone, compared to 6 weeks of conventional therapy with alfacalcidiol 0.25mcg and placebo orally at every dialysis session. The secondary aim is to determine whether this intensive vitamin D supplementation influences or modulates independent activity of the sympathetic and vagal limbs of the cardiac autonomic nervous system compared to the conventional alfacalcidiol regimen.

We hypothesize that vitamin D supplementation will increase *all* cardiac autonomic parameters, but that the vitamin D treatment will specifically have the greatest impact on vagal tone. We hypothesize that cardiosympathovagal balance will decrease after vitamin D therapy due to the fact that any increase in cardiosympathetic tone will be offset by a larger increase in cardiovagal tone.

### Study design and setting

**Figure 1:**
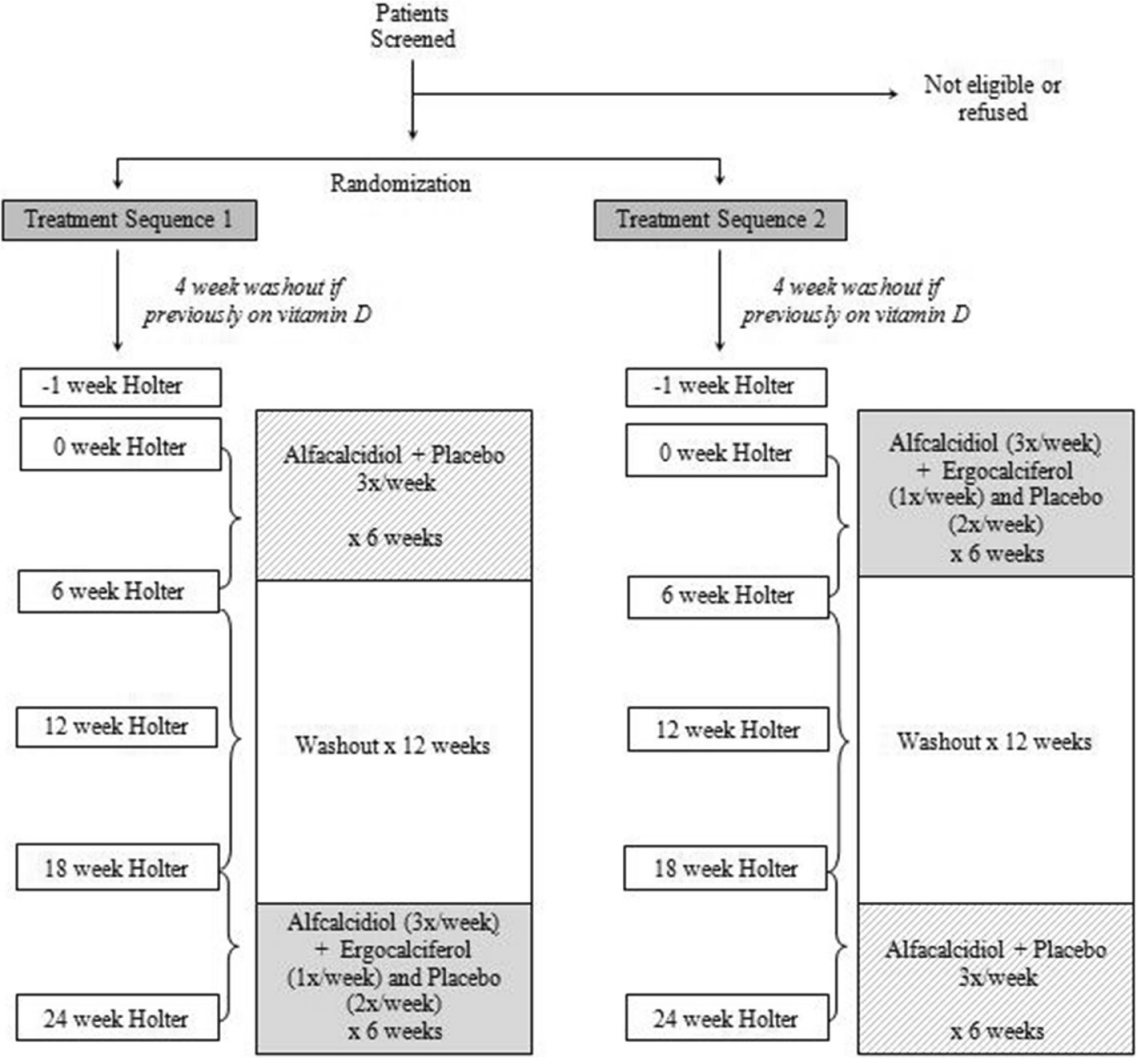
Overview of the VITAH trial.

The VITAH Trial is a 2x2 cross-over, randomized-controlled trial with blinding of subjects, investigators, health care providers, as well as all study coordinators and data analysts. Hemodialysis outpatients from the Southern Alberta Renal Program will be randomized to one of two treatment sequence arms; 6 weeks of intensive vitamin D therapy (0.25 mcg alfacalcidiol at each dialysis session plus 50,000 IU of ergocalciferol once per week and placebo the remaining two weekly dialysis days), followed by a 12 week washout, and 6 weeks of conventional vitamin D therapy (0.25 mcg alfacalcidiol plus placebo at each dialysis session), or vice versa. Twenty-four hour Holter ECG monitors are applied within the first hour of the first weekly dialysis run at baseline and every 6 weeks throughout the entire 24-week duration of the trial (Figure 1). The study protocol will not impact on uremia clearance given that the dialysis prescription will remain constant. Subject recruitment began in January 2013 and will continue until 2016.

### Ethical considerations

Ethical approval has been obtained from the Conjoint Health Research Ethics Board (Project ID: E-24846) at the University of Calgary as well as Health Canada (UC-NEPH-2012001) for all study sites involved in the trial. Two external bodies, a Data Safety Monitoring Board and a Trial Steering Committee, will monitor study progress.

### Study interventions

The intervention is the vitamin D therapy sequence, including 6 weeks of intensive therapy and 6 week of conventional vitamin D therapy, or vice versa (Figure 1). Given that the current clinical standard of care is use of activated vitamin D (one of the top drug classes prescribed as recently outlined in the United States Renal Data System [44]) in the ESKD population, a placebo arm is not included. Upon recruitment, participants who are prescribed any form of vitamin D (either nutritional or active) at that time will cease to take the medication for 4 weeks, a valid washout period employed by the PRIMO trial, a randomized controlled trial examining the effects of activated vitamin D on cardiac structure in subjects with non-dialysis dependent kidney disease [45]. Participants who are not on any form of vitamin D at the time of enrolment will be randomized to one of the two treatment sequences immediately. All participants will be randomized to Treatment Sequence 1 or Treatment Sequence 2, such that each participant will ingest both vitamin D therapy regimens in a randomized, blinded, placebo-controlled crossover design and thus act as his/her own control. A 12 week washout period was chosen between each 6 week vitamin D treatment block to ensure that there are no carryover effects from either study period as the t_1/2_ for alfacalcidiol is approximately 4 hours, and the t_1/2_ for ergocalciferol is 19–48 hours [22,23,46].

### Identification of eligible subjects

Eligible subjects are stable, thrice weekly hemodialysis outpatients within the Southern Alberta Renal Program in Calgary, Alberta. Inclusion and exclusion criteria are outlined in Table 1. All patients in the Southern Alberta Renal Program hemodialysis units will be reviewed. Individuals who meet inclusion criteria will be identified by nephrologists or study investigators. After clearance from the individual’s primary nephrologist, the name will be forwarded to the study coordinators and recorded in recruitment logs. Informed written consent will be obtained from the participant, power of attorney or legal substitute decision-maker. Once eligibility is confirmed by reviewing the chart or from participant interview, baseline clinical (i.e. ESKD etiology, comorbidities, cardiovascular history, dialysis prescription and adequacy details), demographic and medication data will be obtained. Subjects will be randomized 1:1 to one of the two treatment sequences.

**Table 1:** VITAH trial inclusion and exclusion criteria

**Inclusion criteria**	**Exclusion criteria**
• Age ≥ 18 years	• Any major cardiovascular event within the last 6 months prior to enrolment (this includes, but is not limited to, new onset arrhythmia, hospitalization for a cardiovascular complication)
• Thrice weekly hemodialysis outpatient within the Southern Alberta Renal Program	
• Physician consent to participate	• Refusal to cease vitamin D therapy for four (4) weeks prior to initiation of the study
• Ability and agreement to cease any prior vitamin D medication for four (4) weeks prior to initiation of the study	• Palliative status or metastatic malignancy
• Ability to comprehend study protocol and provide oral and written consent in English (native language or translated)	• Known or anticipated upcoming change in dialysis modality including transfer to peritoneal dialysis, kidney transplant, or change in current hemodialysis schedule/duration

### Randomization and blinding

Subject identification codes and corresponding treatment schedules, consisting of two treatment sequences randomized in small random block sizes, were developed by an independent biostatistician at the University of Calgary prior to initiation of the trial. Following informed consent, patients will be given the next available subject identification code and are then randomized to one of the two treatment sequences. The University of Calgary Research Pharmacy is the sole entity that holds the randomization list, and will prepare and dispense blister packs of the appropriate vitamin D supplementation for distribution to each subject accordingly upon email notification of recruitment. The study subjects, investigators, health care providers, study coordinators, and data analysts will be blinded to both the randomization schedule and subsequent treatment allocation.

On treatment days the hemodialysis nurses at each study site will administer the study drug to the subjects at the beginning of each dialysis session throughout the 24-week trial. Compliance with the vitamin D supplementation will be recorded on study drug administration schedules and checklists for each subject enrolled in the trial. To ensure blinding, all vitamin D combinations will be prepared in blister- packages by Research Pharmacy marked only with the participant’s study identification code. Each study capsule is packaged identically in1g soft gel capsules to ensure blinding. Specifically, each study capsule holds one 0.25mcg alfacalcidiol pill as well as a placebo or 50,000 IU ergocalciferol pill. Ergocalciferol has no distinct odour or taste. The Research Pharmacy has manufactured the VITAH placebo pills within each capsule to resemble the ergocalciferol pills in colour, shape, odour, taste and consistency to allow for a blinded controlled trial even if the study capsules are disassembled for the subjects to facilitate ingestion. To ensure compliance with the treatment, all blister-packs will be kept at the respective hemodialysis unit and study medications will be administered and recorded by the dialysis nurse (directly-observed therapy). Ergocalciferol (vitamin D_2_), in contrast to the other commercially available nutritional vitamin D, cholecalciferol (vitamin D_3_), is only available by prescription through a medical vendor and has been labeled with a federal Drug Identification Number through which product can effectively be tracked. This method allows the investigators to determine whether contamination is occurring during the study so it can be reduced and to determine its extent during the analysis phase. Subsequent statistical analyses will also be done blinded to the above treatment allocation.

### Data collection

Ambulatory electrocardiography (Holter) data is recorded for 18–24 hours using a standard bipolar 3-lead configuration (GE Healthcare, SEER MC; Milwaukee, USA). HRV is analyzed using a commercial Holter analysis system (MARS v. 7; GE Healthcare; Milwaukee, USA). Power spectral density analysis, which transforms the electrocardiographic signals into measures of frequency domain HRV (representing activity of the cardiac autonomic nervous system) are calculated. Autonomic activity is categorized into spectral bands: total power (TP), very-low frequency (VLF, 0.003-0.04 Hz), low-frequency (LF, 0.04-0.15 Hz) and high-frequency (HF, 0.15-0.4 Hz) domains [7,8,17-19]. Absolute LF and HF parameters are then squared and log-transformed (*ln* ms [2]) and converted to normalized units (nu). Overall cardiosympathovagal balance (LF:HF) is derived from these measures. Time domain HRV measurements are also calculated (Table 2) [7,8,17-19].

**Table 2:** Description of heart rate variability data to be analyzed from 24- hour Holter recording

**Heart rate variability parameter (Acronym)**	**Description/Physiological representation**
**Low frequency (LF)**	Represents contribution of baroreflex activity in overall cardiovascular control
**High frequency (HF)**	Represents contribution of cardiac parasympathetic/vagal nervous activity
**Low to high frequency ratio (LF:HF)**	Represents the interplay between sympathetic and parasympathetic limbs of the autonomic nervous system
**Standard deviation of the normal**	
**Wave (SDNN)**	Variation (in units of standard deviation) between each successive R-wave in the ECG recording
**Standard deviation of the average**	
**Normal wave (SDANN)**	Average of the variation (in units of standard deviation) between each successive R-wave when comparing multiple 5-minute sections of the ECG recording
**Percentage of normal wave variation (pNN50%)**	Percentage of normal R-waves that differ from the wave directly before it by ≥50 milliseconds

The specific physiological parameters which will be assessed at each study visit throughout the study are described in Table 3. A MEDRIO electronic database (http://www.medrio.com) will be utilized for data entry after collection at baseline and every 6 weeks up to 24 weeks. Holter application and simultaneous blood draws will be collected at each study visit in order to observe potential vitamin D therapy-dependent changes in cardiac autonomic tone, circulating renin-angiotensin system activity markers, and mineral metabolism parameters related to vitamin D metabolism including serum calcium, phosphate, and parathyroid hormone. Blood samples collected from subjects at each study day are sent to the on-site laboratory typically used for other routine blood work at each of the study locations. Database access and ability to update blinded data will be provided only to the primary investigators.

**Table 3:** Baseline and ongoing data collection

**Screening visit**	• Age, sex, target weight, duration on dialysis, cause of end-stage kidney disease, vascular access type
	• Serum calcium, phosphate, parathyroid hormone, Kt/V, 25-hydroxy vitamin D, 1,25-dihydroxy vitamin D
	• Medication use
	• GODIN leisure-time activity questionnaire
	• DASI aerobic capacity questionnaire
**Baseline study visit**	• Serum calcium, phosphate, parathyroid hormone, Kt/V, 25OHD level, 1,25-dihydroxy vitamin D level, catecholamines
	• Renin, aldosterone, angiotensin II
	• Delivered dose of dialysis (Kt/V)
	• Power spectral analysis (LF, HF, LF:HF) and time domain (SDNN, SDANN, pNN50%) HRV parameters
**Study visits every 6 weeks up to 24 weeks**	• Serum calcium, serum phosphate, parathyroid hormone, 25OHD, 1,25-dihydroxy-vitamin D, catecholamines
	• Renin, aldosterone, angiotensin II
	• Delivered dose of dialysis (Kt/V)
	• Power spectral analysis (LF, HF, LF:HF) and time domain (SDNN, SDANN, pNN50%) HRV parameters

### Primary outcome

The primary outcome of the study is sympathovagal balance (LF:HF), interpreted as the overall balance on stimulatory and inhibitory cardiac autonomic control, from baseline to the final study day within each 6 week treatment block (0 and 6 weeks, 18 and 24 weeks). Comparison of measured sympathovagal balance throughout each of the vitamin D supplementation treatment blocks as well as the 12-week washout period will allow for statistical interpretation of whether presence or absence of oral vitamin D therapy alters cardiac autonomic function within this population. An improvement in sympathovagal balance can be interpreted by a downward shift in LF:HF, in which a smaller value indicates greater contribution of cardioprotective vagal activity (Table 2).

### Secondary outcomes

The secondary outcomes include additional measures of cardiac autonomic tone, including LF, HF, as well as time domain parameters of HRV including the standard deviation of the normal R-R interval (SDNN), standard deviation of the average normal R-R interval (SDANN), percentage of normal R waves occurring ≥ 50 ms after the R waves immediately before (pNN50%), as well as blood draw measurements related to vitamin D metabolism at the beginning and termination of each 6 week treatment block (Table 2).

### Subject follow-up procedures

Subjects will be followed from trial entry until study completion (24 weeks study period). The study coordinator will review each subjects’ collected data at 6 week intervals to record primary and secondary endpoints, as well as to assess the safety and efficacy of the vitamin D administration in relation to study blood work. Study medication may be discontinued for reasons associated with concern for subject safety, or as requested by the subject or primary nephrologist.

### Study withdrawal

Given that the primary and secondary outcomes require the measurement of cardiac autonomic tone with a Holter monitor, follow up for these outcomes can only occur if the subject is alive and accessible for monitoring. Therefore, subjects will be censored and withdrawn from follow-up under the following circumstances; subject or primary nephrologist request, transfer to peritoneal dialysis, kidney transplantation, transfer of subject to a non-study hemodialysis unit, hospitalization, or death. Upon withdrawal from the study, any data collected up to that time will be utilized in a last observation carried forward (LOCF) approach.

### Statistical analysis

The primary analysis will test associations between intensive and conventional vitamin D therapies and observed fluctuations in LF:HF from baseline to 6 weeks later within each treatment block utilizing a non-parametric paired t-test. To further determine the relationships between cardiac autonomic tone and specific vitamin D therapy regimens, we will use a repeated measurements analysis of variance model to account for the cross-over design of the study utilizing Greenhouse-Geisser correction values to account for multiple comparisons. Treatment, treatment period, and treatment sequence will be analyzed as fixed effects within the model, as well as additional effect modifiers (gender, vitamin D, calcium, phosphate, parathyroid hormone). Additional analyses will include a subgroup analysis for those subjects with diabetes mellitus as diabetes has been shown to alter parameters of cardiac autonomic tone and vitamin D metabolism [9,21].

### Sample size calculation

While we are not aware of any studies examining the use of vitamin D on the primary outcome of this study, previous work by Kontopoulous et al. [47] has demonstrated that post-myocardial infarction (MI) patients (n = 25) randomized to receive 5–10 mg of quinapril (angiotensin converting enzyme inhibitor) per day for 35 days immediately following an MI displayed a 46% reduction in LF:HF from baseline to 6 weeks (mean ± SE: 10 ± 0.7; 5 ± 0.5), while those randomized (n = 25) to receive 50–100 mg of metoprolol per day for 35 days following an MI displayed a similar 36% reduction in LF:HF from baseline to 6 weeks (mean ± SE: 9 ± 0.6; 6 ± 0.4). As such, given that 1) vitamin D deficiency is the norm in hemodialysis patients, 2) vitamin D downregulates the renin-angiotensin system [24,25,35], and 3) increased RAS activity is associated with alterations in cardiac autonomic tone [35-37], we feel there is sufficient justification for the sample size calculation herein**.** Based on these observations, we estimate the standard deviation of the pooled difference in LF:HF from both treatment sequences as 4.53. Using a 2 × 2 crossover design (2 sequences or treatment orderings and 2 time periods or occasions) and anticipating a 20% or larger reduction in LF:HF observed after 6 weeks of treatment with combined vitamin D vs. the reduction observed after 6 weeks of treatment with alfacalcidiol alone, we estimate that 54 subjects will be required (α = 0.05, β = 0.9). In anticipation of a 10% dropout rate, approximately 60 subjects in total will be recruited.

## Discussion

In summary, the VITAH trial will determine whether an intensive vitamin D supplementation regimen will improve cardiac autonomic tone compared to conventional vitamin D supplementation in patients receiving chronic hemodialysis. As the trial will remain relatively small in nature, it is possible that potential sources of bias may influence the results obtained. For example, subjects recruited must be stable dialysis patients which may represent a different area of the patient spectrum in terms of the benefit that may be derived from vitamin D supplementation therapy. Furthermore, our study is limited to the Calgary, Alberta area in Canada which may play a role in the vitamin D levels of our subject population based solely on geographical location and seasonal sunlight exposure although current evidence would suggest that a majority of dialysis patients are vitamin D deficient regardless of geographical location [24,26-28]. The evidence surrounding the relationship between various vitamin D therapies and cardiovascular risk derived from clinical trials is extremely scarce. The OPERA study by Wang and colleagues [48] has recently demonstrated that in subjects with non-dialysis dependent chronic kidney disease, 52 weeks of daily paricalcitol supplementation did not alter left ventricular mass, volume, or ejection fraction. Critically, the study showed that cardiovascular-related hospitalization rates were significantly reduced in the vitamin D treatment group compared to placebo, suggesting that vitamin D may affect cardiovascular risk and underscoring the need for other cardiac parameters, such as cardiac autonomic tone, to be evaluated in relation to vitamin D supplementation. The VITAH trial will lend much needed data to the literature pool, and moreover conclusions drawn from this trial will provide quality evidence towards the current equipoise which exists within the discussion of the clinical efficacy of vitamin D in reducing cardiovascular-specific risk in this growing vulnerable chronic patient population.

## Abbreviations

25OHD: 25-hydroxyvitamin D; ESKD: End-stage kidney disease; HRV: Heart rate variability; HF: High-frequency; LOCF: Last observation carried forward; LF: Low-frequency; LF:HF: Low-frequency: High-frequency; MI: Myocardial infarction; PNS: Parasympathetic nervous system; SDNN: Standard deviation of the normal R-R interval; SDANN: Standard deviation of the average normal R-R interval; SCD: Sudden cardiac death; SNS: Sympathetic nervous system; TP: Total power; VLF: Very-low frequency.

## Competing interests

This work was supported by an Establishment Grant from Alberta Innovates – Health Solutions, the University of Calgary Faculty of Medicine, and the University of Calgary Division of Nephrology. Ms. MC Mann is supported by the Canadian Institute of Health Research (CIHR) Doctoral Research Award as well as the University of Calgary Achievers in Medical Science Graduate Recruitment Scholarship. Drs. DV Exner, BR Hemmelgarn, and SB Ahmed are supported by Alberta Innovates – Health Solutions and Canadian Institutes of Health Research. Dr. DV Exner is the Canada Research Chair in Cardiovascular Clinical Trials and receives unrestricted support from GE Healthcare. Funding sources had no role in the design, conduct, or reporting of this study. The authors declare no conflict of interest.

## Authors’ contributions

MCM and SBA were responsible for identifying the research question and drafting the study protocol. All authors have contributed to the development of the protocol and study design, as members of the research team. MCM and SBA were responsible for drafting of this paper and all authors provided comments and have read and approved the final version.

## Pre-publication history

The pre-publication history for this paper can be accessed here:

http://www.biomedcentral.com/1471-2369/15/129/prepub
